# Magnitude of multiple drug use and determinants of vulnerability among chronic kidney disease inpatients in Ethiopia: a multi-center study

**DOI:** 10.1186/s12882-024-03773-x

**Published:** 2024-10-07

**Authors:** Tirsit Ketsela Zeleke, Rahel Belete Abebe, Samuel Agegnew Wondm, Bantayehu Addis Tegegne

**Affiliations:** 1 Department of Pharmacy, College of Health Sciences, Debre Markos University, Debre Markos, Ethiopia; 2 Department of Clinical Pharmacy, School of Pharmacy, College of Medicine and Health Sciences, University of Gondar, Gondar, Ethiopia

**Keywords:** Multiple drug use, Chronic kidney disease, Ethiopia

## Abstract

**Background:**

Patients with chronic kidney disease frequently face various nutritional and metabolic problems that necessitate the use of multiple medications. This multiple drug use can lead to several drug-related problems including adverse drug events, hospital admissions, poor medication adherence, harmful drug interactions, inadequate therapeutic outcomes, and death. Despite these challenges, there is a notable lack of studies on the extent of multiple drug use and its determinants among patients with chronic kidney disease in Ethiopia. This study aims to assess the magnitude of multiple drug use and identify the determinants of vulnerability among patients with chronic kidney disease in Ethiopia.

**Method:**

A hospital-based cross-sectional study was conducted among patients with chronic kidney disease. Eligible participants were selected using a simple random sampling technique. Frequency and percentage calculations were performed for categorical variables, while means and standard deviations were used for continuous variables. The chi-square test and t-test were used to compare the proportions and means, respectively. Binary logistic regression was used to identify the determinants of multiple drug use, with statistical significance determined by a p-value of less than 0.05 and a 95% confidence interval. Guidelines and previous literature were utilized to assess the magnitude of multiple drug use.

**Results:**

A total of 230 patients were enrolled, with more than half being male. The overall magnitude of multiple drug use was 83.0%. Diuretics being the most frequently prescribed medication class followed by angiotensin converting enzyme inhibitors. Patients aged 65 years and above (AOR = 4.91 (95% CI 1.60-15.03)), CKD stage five (AOR) = 5.48 (95% CI 1.99–15.09)), and the presence of comorbid conditions (AOR) = 3.53 (95% CI 1.55–8.06)) were significantly associated with multiple drug use.

**Conclusion:**

Chronic kidney disease patients exhibited a high rate of multiple drug use. The presence of comorbid conditions, disease progression and older age are significant determinates of this vulnerability. Health care providers should pay particular attention to these factors to manage and mitigate the risks associated with multiple drug use.

## Introduction

Chronic kidney disease (CKD) is defined as diminished kidney function, regardless of the underlying cause, indicated by a creatinine clearance (glomerular filtration rate) of less than 60 mL/min per 1.73 m² or the presence of kidney injury indicators, or both, for a minimum of three months [1]. It is a global public health issue that affects over 800 million individuals worldwide [2] and is associated with increased healthcare costs and unfavorable outcomes [3]. CKD patients experience a wide range of nutritional and metabolic problems, leading to a significant degree of complications and comorbidities [4]. Patients with CKD often present with multiple comorbidities, such as anemia, hypertension, cardiovascular disease, bone and mineral disease, and diabetes [5]. Consequently, they typically require a wide range of medications and complex drug regimens to manage the disease, slow its progression, and treat comorbid conditions [6, 7]. This need introduces multiple drug use into their daily lives [8, 9]. “Multiple drug use”, or polypharmacy is defined as the concurrent use of at least five medications [10, 11]. The pharmacological management of CKD and its related comorbidities is challenging due to the need for multiple medications and appropriate dose adjustments [12]. Furthermore, the presence of CKD makes the risk-benefit ratio for many medications uncertain [9]. CKD can affect several organ systems, leading to significant alterations in the pharmacokinetics and pharmacodynamics of many drugs, which increases the risk of medication-related problems such as drug interactions and adverse drug reactions, exacerbating the difficulties already faced by CKD patients [13–16]. CKD patients with multiple medications are at high risk for drug-related problems, including adverse drug events, hospital admissions, poor medication adherence, harmful drug interactions, inadequate therapeutic outcomes, and potentially preventable death [17, 18]. This situation poses significant clinical and economic challenges for patients, communities, and the healthcare sector [19]. Clinicians can develop and implement interventions, such as reducing prescriptions and deprescribing, to minimize the number of medications and their harmful effects by understanding the magnitude of multiple drug use among CKD patients and identifying vulnerable populations [20].

Despite the well-known challenges of drug therapy in CKD patients, there is limited information about the current practice of multiple drug use, its magnitude, and its determinants among CKD patients in Ethiopia. The aim of this study was to assess the extent of multiple drug use and identify factors contributing to vulnerability among CKD patients in Ethiopia.

## Methods

### Study setting and design

A hospital-based cross-sectional study was conducted from July 30, 2022, to February 10, 2023, at three specialized comprehensive hospitals in northwest Ethiopia: Debre Markos Comprehensive Specialized Hospital, Felege Hiwot Comprehensive Specialized Hospital, and University of Gondar Comprehensive Specialized Hospital.

### Source and study population

The source population comprised all adult CKD patients admitted to the three hospitals, while the study population included those CKD patients available during the data collection period.

### Eligibility criteria

Patients aged 18 years and older who were on at least one medication were eligible for the study. Patients who were unable to communicate or had incomplete clinical documentation were excluded. Participants were required to provide written informed consent before participating in the study. This consent process ensured that participants were fully informed about the nature of the study, its objectives, and other ethical considerations.

### Sample size determination

Single population proportion formula was used to estimate the sample, where the following assumptions were considered: 50% prevalence, 95% confidence interval, and 5% margin of error.

- $\:n=\frac{{\left(\text{Z}\text{a}2\right)}^{2}p\left(q\right)}{{d}^{2}}$, where Z statistics (at 95% CI= 1.96), p (population proportion), and d (margin of error)

$$n = {{{{\left( {1.96} \right)}^2} \times 0.5 \times 0.5} \over {{{0.05}^2}}} = 384$$

However, based on the previous three hospital’s report of CKD cases, the total number of patients who presented annually were 571, which was less than 10,000. Hence, we used a population correction formula to determine the final sample size. As a result, we calculated our final sample size using a population correction.

- Population correction formula.

$$\:nf=\frac{n}{1+\frac{n}{N}}$$

Where:

- N = Total number of patients who could be presented.
- n = Sample size before population correction.
- nf = Final sample size.

$$\:nf=\frac{384}{1+\frac{384}{571}}=230$$

Therefore, our final sample size was **230**.

### Sampling technique

Simple random sampling technique was used to select three hospitals for the study. Proportional allocation was then used to enroll patients from each hospital based on their respective patient populations. Within each hospital, a simple random sampling procedure was utilized to choose study participants.

### Study variables

Multiple drug use was the dependent variable. While sociodemographic parameters (sex, age, residence, educational level, occupation, monthly income), drug-related parameters (name, dose, and frequency of drug use), medical-related parameters (existence of comorbid conditions, stage of the disease, type/s of the comorbid conditions, presence of dialysis), and practitioner specialty were included as independent variables.

### Operational definition

- **Multiple drug use** is defined as the concomitant use of at least five medications [10, 11].
- **Index Drug Therapy/Index Multidrug Therapy**: Refers to the use of a primary drug or combination targeting the main pathology of CKD.
- **Co-Drug Therapy**: The concurrent use of two or more drugs targeting different aspects of CKD or its comorbid conditions to achieve synergistic effects.
- **Multidrug Therapy**: The comprehensive use of multiple drugs to manage the multifaceted nature of CKD and its associated complications.
- **Stage 1 CKD**: Characterized by kidney damage with a normal or increased glomerular filtration rate (GFR ≥ 90 mL/min/1.73 m²).
- **Stage 2 CKD**: Defined as kidney damage with a mild decrease in GFR (60–89 mL/min/1.73 m²), similar to Stage 1 but with a reduced GFR.
- **Stage 3 CKD** indicates a moderate decrease in GFR (30–59 mL/min/1.73 m²).
- **Stage 4 CKD** involves a severe decrease in GFR (15–29 mL/min/1.73 m²), reflecting a significant reduction in kidney function.
- **Stage 5 CKD**: Also known as end-stage renal disease, is characterized by kidney failure (GFR < 15 mL/min/1.73 m²).

### Data collection tools and procedure

Following a review of relevant literature, the authors designed a structured questionnaire, which was provided to data collectors to extract data [3, 19–22]. The questionnaire was initially developed in English by the investigator. To ensure uniformity, it was subsequently translated into Amharic and then back-translated into English by bilingual staff. Necessary modifications were made based on this translation to refine the instrument. The study’s principal investigator provided training to supervisors and data collectors, covering the study’s objectives, methodology, and ethical considerations.

The validation of questionnaire involved a multi-step process. Initially, the first version of the questionnaire was created based on a comprehensive review of relevant literature and existing validated instruments, ensuring that the content was thorough and aligned with the study’s objectives. The draft questionnaire was reviewed by a panel of experts, including nephrologists, clinical pharmacists, and nurses, to assess its content and face validity. These experts provided valuable feedback on the relevance, clarity, and comprehensiveness of the questions as well as its appearance, understandability, and suitability for the target population. The panel reached a consensus that the questionnaire demonstrated strong content validity and adequately covered all necessary aspects of multiple drug use in patients with CKD.

Six nurses and two supervisors participated in the data collection process. The data collection instrument was divided into three parts: sociodemographic parameters of the participants, medical-related parameters, and drug related parameters. Clinical findings and prescribed medications were retrieved from medical records, while sociodemographic information was obtained through interviews. Regarding the assessment of the presence of comorbid conditions, self-report and medical records were used in combination. Self-described comorbidities were evaluated using medical records and cross-checked with those listed in their medical documentation. A list of comorbidities was selected based on their wide range of availability in individuals with CKD and their straightforwardness of detection from medical data and patient self-report. The comorbid conditions chosen consisted of cerebrovascular illness (stroke), hypertension, anxiety, depression, or both (or other mental health issues), dyslipidemia, heart disease (heart attack, cardiac arrhythmias, ischemic heart disease, atrial fibrillation), diabetes, peripheral vessel diseases, muscular and skeletal diseases, malignancy, hepatic disease, and others.

Monitoring parameters, such as weight, were not usually recorded in the medical charts; in such cases, the findings were obtained through observations and recorded accordingly. Laboratory tests, including serum creatinine levels, were also taken from the patients’ medical charts, and the estimated creatinine clearance was calculated using the Cockcroft-Gault formula [23]. The stages of CKD were classified according to the Kidney Disease: Improving Global Outcomes (KDIGO) guideline. CKD patients who met the eligibility criteria after hospitalization were included in the study. The assessment of multiple drug use has been done based upon previous guidelines and literature [10, 11].

### Data processing and analysis

After coding, checking for accuracy and consistency, and imported into Epi-data 4.6, the data were exported to SPSS 26 for more analysis. Frequency and percentage calculations were performed for categorical variables (such as sex, residence, education, occupation, presence of comorbidities, type of comorbid conditions, stage of the disease, presence of dialysis, and practitioner specialty), while the mean and standard deviation were calculated for continuous variables (such as age, weight, serum creatinine, creatinine clearance, number of medications, and monthly income). The chi-square test and the t-test were used to compare the proportions and means, respectively. A binary logistic regression model was used to analyze the determinates of multiple drug use. The Hosmer and Lemeshow goodness-of-fit test assessed the model’s fitness. The multicollinearity of the independent variables was analyzed using the variance inflation factor, which was within an acceptable level of one to five, to identify and eliminate redundant variables could affect our estimates. Variables with a p-value less than 0.25 in the bivariable analysis were entered into the multiple binary logistic regression model. The statistical association was reported using the adjusted odds ratio, with a p-value of < 0.05 indicating a significant association with the outcome variable. The study results were illustrated using texts, figure, and tables.

### Data quality assurance

we employed the Content Validity Index (CVI), a well-established statistical measure used to assess the relevance, clarity, and comprehensiveness of questionnaire items. A panel of five experts in clinical pharmacy and nephrology evaluated each item in the questionnaire. The Item-Level Content Validity Index (I-CVI) for each item was calculated by dividing the number of experts who rated the item as “relevant” or “very relevant” by the total number of experts. Items with an I-CVI of 0.78 or higher were considered acceptable. For the overall scale, the Scale-Level Content Validity Index (S-CVI) was calculated by averaging the I-CVI values across all items, with a threshold of 0.90 used to indicate excellent content validity. Our analysis revealed that the I-CVI values ranged from 0.80 to 1.00, demonstrating strong relevance and clarity across the questionnaire items. The S-CVI was calculated to be 0.92, indicating excellent overall content validity.

To further evaluate the questionnaire’s clarity and sociocultural appropriateness, and feasibility of the administration process, a pretest was conducted at Tibebe Ghion Specialized Hospital with 12 chronic kidney disease inpatients (representing 5% of the total sample). Feedback from the pretest indicated that the questionnaire was clear and understandable, with no major issues reported by the participants.

To ensure data quality, the investigator meticulously checked the collected data for completeness and consistency immediately upon entry. Automated validation rules and manual cross-checks were implemented during the data entry process to identify and rectify any missing or inconsistent entries. A systematic process for data verification was established, involving regular audits to assess data quality and consistency. Any discrepancies were promptly addressed through follow-up with data collectors to ensure accuracy and completeness. Supervisors provided on-site supervision throughout the data collection period, ensuring adherence to protocols and facilitating the immediate resolution of any issues that arose.

## Results

### Sociodemographic related parameters of the study participants

A total of 230 patients with a 100% response rate were enrolled in the study. The majority of patients were between 50 and 64 years old, and their average weight was 60.92 kg. More than half of the participants (54.8%) were male, and 52.2% resided in rural areas. Regarding education, 28.7% of the participants had a college or higher education level, while 29.6% were employed by the government. The average monthly income was 47.96 United States Dollar (USD) (Table 1).

**Table 1:** Sociodemographic parameters of the study participants

	Variables		Frequency (*n*)		Percent (%)		
Age							
	20–34		48		20.9		
	35–49		46		20.0		
	50–64		71		30.9		
	≥ 65		65		28.3		
Sex							
	Male		126		54.8		
	Female		104		45.2		
Residence							
	Rural		120		52.2		
	Urban		110		47.8		
Education status	No formal education		63		27.4		
	Primary education		40		17.4		
	Secondary education		61		26.5		
	College and above		66		28.7		
Occupation							
	Farmer		61		26.5		
	Housewife		34		14.8		
	Government employee		68		29.6		
	Private worker		57		24.8		
	Other*		10		4.3		
			Mean		Standard deviation		
Monthly income in USD		47.96		± 32.84			

* pensioner, daily laborer, student USD United States Dollar

### Medical related parameters of the study participants

Regarding the stage of CKD, 27.8% of the patients were in stage five. Most of the participants (72.6%) had comorbid conditions. Hypertension was the most common comorbid condition, occurring in 59.5% of the patients, followed by diabetes mellitus in 42.6%. Laboratory recordings showed that the median serum creatinine level was 2.10 mg/dl, while the median creatinine clearance was 32.84 mL/min. Moreover, 24.8% of the patients were undergoing dialysis treatment. More than half of the prescriptions (55.2%) were made by general practitioners (Table 2).

**Table 2:** Medical related parameters of the study participants

	Variable	Frequency (*n*)	Percent (%)
Stage of the disease			
	Stage one	52	22.6
	Stage two	36	15.7
	Stage three	35	15.2
	Stage four	43	18.7
	Stage five	64	27.8
Dialysis therapy			
	Absent	173	75.2
	Present	57	24.8
Comorbid condition			
	Absent	63	27.4
	Present	167	72.6
	Type/s of comorbid conditions		
	Hypertension	137	59.5
	Diabetes mellitus	98	42.6
	Congested heart failure	73	31.7
	Anemia	69	30.0
	Urinary tract infection	51	22.2
	Acute glomerulonephritis	42	18.3
	Sepsis	21	9.1
	Others*	13	5.6
	Practitioner specialty		
	General physician	127	55.2
	Internist	103	44.8
		Mean	Standard deviation
Weight		60.92 kg	± 10.41
Number of drugs per patient		6.47	2.20
		Median	Interquartile range
Serum creatinine level		2.10 mg/dl	1.90 mg/dl to 4.40 mg/dl
Creatinine clearance		32.84 mL/min	20.00 mL/min to 74.15 mL/min

*asthma, glaucoma, arthritis, deep venous thrombosis, pneumonia

### Magnitude of multiple drug use and prescription pattern

A total of 27 types of medications were prescribed, with an average of 6.47 drugs per patient. Diuretics were the most frequently prescribed class of drugs, followed by angiotensin converting enzyme inhibitors. Regarding specific drug prescriptions. Furosemide was the most frequently prescribed medication (87.4%), followed by enalapril (85.6%), amlodipine (48.7%), spironolactone (43.9%), and cimetidine (42.2%).

In analyzing medication prescription frequency among CKD patients on dialysis therapy versus those not on dialysis, 57 patients on dialysis and 173 patients not on dialysis were included. Furosemide was the most commonly prescribed medication among both groups, with 50.9% of patients on dialysis therapy and 99.4% of those not on dialysis receiving it, resulting in a total prescription rate of 87.4%. Enalapril was prescribed to 43.8% of patients on dialysis and 95.3% of patients not on dialysis, making it the second most frequently prescribed medication overall, with a total rate of 85.6% (Table 3).

**Table 3:** Frequency of medication prescriptions among the study participants

	Variable	Frequency of prescription per patient		
Name of medication		On Dialysis therapy (57)	Not on Dialysis therapy (173)	Total prescription
	Furosemide	29 (50.9%)	172 (99.4%)	201 (87.4%)
	Enalapril	25 (43.8%)	172 (99.4%)	197 (85.6%)
	Amlodipine	14 (24.6%)	98 (56.6%)	112 (48.7%)
	Spironolactone	5 (8.8%)	96 (55.5%)	101 (43.9%)
	Hydrochlorothiazide	0 (%)	92 (53.2%)	92 (40.0%
	Cimetidine	9 (15.8%)	88 (50.9%)	97 (42.2%)
	Insulin	7 (12.3%)	60 (34.7%)	67 (29.1%)
	Nifedipine	11 (19.3%)	42 (24.3%)	53 (23.0%)
	Aspirin	4 (7.0%)	31 (18.0%)	35 (15.2%)
	Atorvastatin	6 (10.5%)	33 (19.1%)	39 (16.9%)
	Ceftriaxone	20 (35.1%)	53 (30.6%)	73 (31.7%)
	Omeprazole	18 (31.6%)	41 (23.7%)	59 (25.6%)
	Metoprolol	12 (21.1%)	26 (15.0%)	38 (16.5%)
	Metformin	0 (%)	12 (5.8%)	12 (5.2%)
	Ciprofloxacin	5 (8.8%)	22 (12.7%)	27 (11.7%)
	Metronidazole	9 (15.8%)	39 (22.5%)	48 (20.9%)
	Ferrous sulphate	16 (28.1%)	48 (27.7%)	64 (27.8%)
	Ceftazidime	21 (36.8%)	50 (28.9%)	71 (30.9%)
	Vancomycin	19 (33.3%)	50 (28.9%)	69 (30.0%)
	Atenolol	3 (5.3%)	18 (10.4%)	21 (9.1%)
	Others*	2 (1.8%)	10 (6.9%)	13 (5.6%)

*beclomethasone, prednisolone, acetazolamide, clopidogrel, warfarin, heparin, meloxicam

**Fig. 1:**
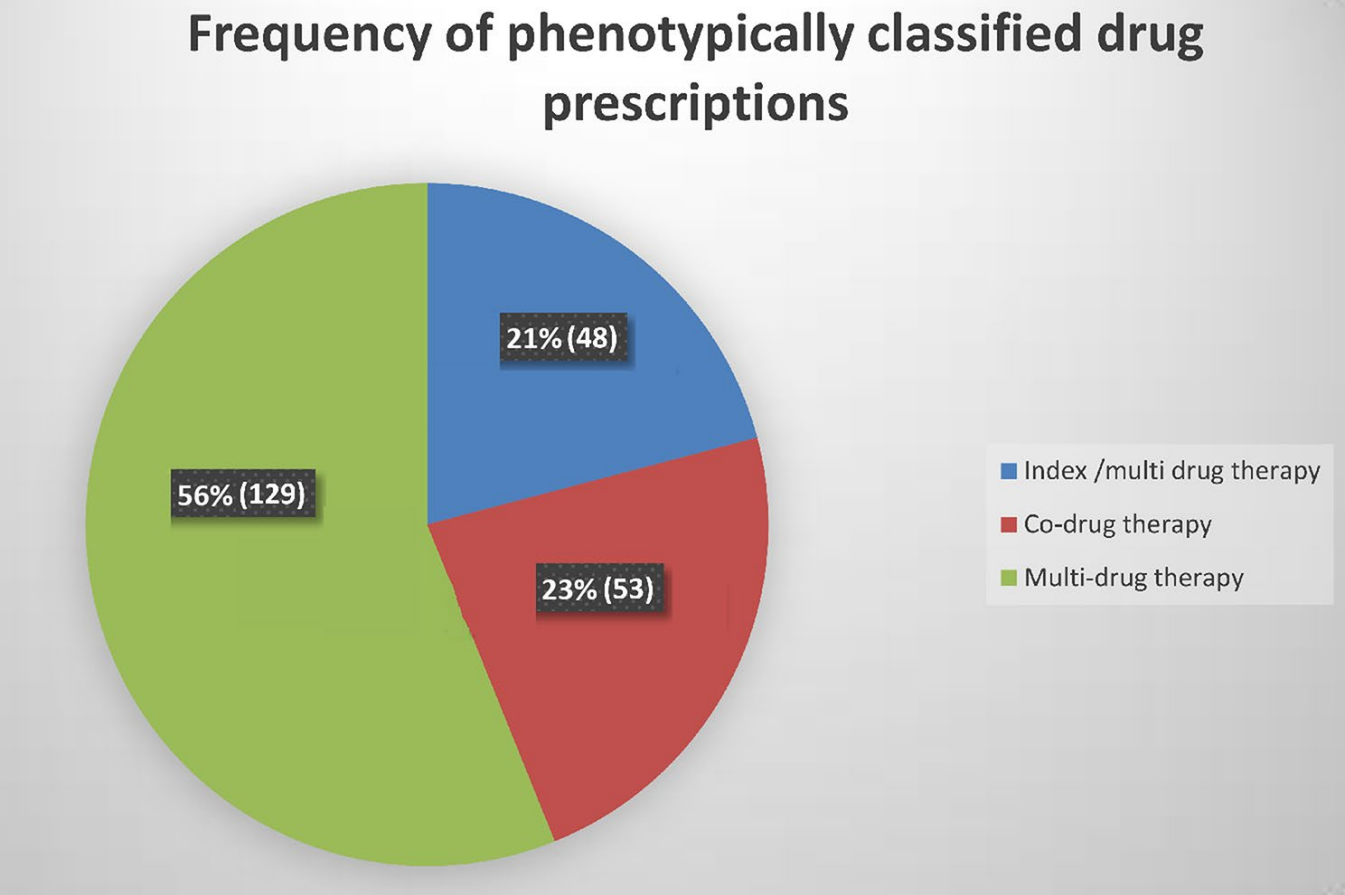
Frequency of phenotypically classified drug prescriptions among study participants

Among the 230 patients in the study, 191 (83.0%) were prescribed multiple medications, defined as the use of at least five different medications according to our operational definition. This high prevalence reflects the typical clinical practice for CKD patients, who often require a range of drugs to manage their condition and associated comorbidities. The remaining 39 patients (17.0%) were prescribed fewer medications. Regarding the three phenotypic classes of drug therapies, the majority of patients (56.1%) used multidrug therapy (Fig. 1).

### Distribution of multiple drug use among different groups

Chi-square and t-test analyses revealed no significant differences in multiple drug use across different groups based on sex, residence, education status, occupation, dialysis therapy, and practitioner specialty. However, significant differences were observed in multiple drug use among patients with comorbid conditions, older age, and different stages of the disease. Among patients without comorbid conditions, 69.8% were on multiple drug therapy, whereas 30.2% were not. In contrast, 88.0% of those with comorbid conditions were on multiple drug therapy, and 12.0% were not. The chi-square test result was χ² = 10.740, *p* = 0.001, indicating a significant difference in multiple drug use between patients with and without comorbid conditions. In stage one, 59.6% of patients were on multiple drug therapy compared to 40.4% not on it. For stage two, 88.9% were on multiple drug therapy and 11.1% were not. Stage three showed 91.4% on multiple drug therapy and 8.6% not on it. In stage four, 90.7% were on and 9.3% were not on multiple drug therapy. Finally, in stage five, 89.1% were on multiple drug therapy compared to 10.9% not on it. The chi-square test result was χ² = 26.326, *p* < 0.001, indicating a significant difference in multiple drug use across different stages of the disease. More over The percentage of patients on multiple drug therapy increases with age, with the highest proportion in the oldest age group (≥ 65 years). The Chi-square test reveals a significant association between age and multiple drug use (X² = 11.932, *p* = 0.008) (Table 4).

**Table 4:** Distribution of multiple drug use among different groups

	Variable	Multiple drug use				X^2^ test	*P* value
		Yes		No			
		Frequency (n)	Percent (%)	Frequency (n)	Percent (%)		
Age							
	20–34	32	66.7	16	33.3	11.932	**0.008**
	35–49	39	84.8	7	15.2		
	50–64	62	87.3	9	12.7		
	≥ 65	58	89.2	7	10.8		
Sex							
	Male	110	87.3	16	12.7	3.588	0.058
	Female	81	77.9	23	22.1		
Residence							
	Rural	96	80.0	24	20.0	1.650	0.199
	Urban	95	86.4	15	13.6		
Education status							
	No formal education	54	85.7	9	14.3	1.395	0.707
	Primary level	31	77.5	9	22.5		
	Secondary level	50	82.0	11	18.0		
	College and higher level	56	84.8	10	15.2		
Occupation							
	Farmer	50	82.0	11	18.0	1.465	0.833
	House wife	29	85.3	5	14.7		
	Government employee	57	83.8	11	16.2		
	Private worker	48	84.2	9	15.8		
	Other	7	70.0	3	30.0		
Stage of the disease							
	Stage one	31	59.6	21	40.4	26.326	< 0.001*
	Stage two	32	88.9	4	11.1		
	Stage three	32	91.4	3	8.6		
	Stage four	39	90.7	4	9.3		
	Stage five	57	89.1	7	10.9		
Comorbid condition							
	Absent	44	69.8	19	30.2	10.740	0.001*
	Present	147	88.0	20	12.0		
Dialysis therapy							
	Absent	143	82.7	30	17.3	0.073	0.787
	Present	48	84.2	9	15.8		
Practitioner specialty							
	General physician	107	84.2	20	15.8	0.294	0.588
	Internist	84	81.5	19	18.5		

*Statically significant

### Determinants of vulnerability to multiple drug use

The findings from the binary logistic regression analysis showed that sociodemographic parameters such as sex, residence, education, occupation, and monthly income, medical-related parameters including stage of the disease, and dialysis therapy did not show a significant association with multiple drug use, while being older, CKD stage progression, and the existence of comorbid conditions were determinates of multiple drug use. Participants who presented with a comorbid condition had a 3.53 times greater risk of receiving multiple drugs compared to those who had no comorbid condition (AOR = 3.53 (95% CI 1.55–8.06)). Patients in stages two through five are at a considerably higher risk of using multiple drugs, compared to those in stage one, with AOR of 5.99 (95% CI 1.67–21.43)) for stage two, 6.49 (95% CI 1.63–25.87 )) for stage three, 6.36 (95% CI 1.82–22.26 )) for stage four, and 5.99 (95% CI 1.99–15.09)) for stage five, respectively. The other significantly associated variable was age; Individuals aged 50 to 64 are 4.21 times more risk of multiple drug use compared to younger individuals (AOR = 4.21, 95% CI 1.45–12.25). Similarly, those aged 65 and above have a 4.91 times higher risk of multiple drug use compared to younger individuals (AOR = 4.91, 95% CI 1.60-15.03) (Table 5).

**Table 5:** Determinants of multiple drug use among the study participants

	Variables	Multiple drug use		Bivariable analysis			Multi variable analysis		
		Yes	No	P-value	COR	CI	P- value	AOR	CI
Age									
	18–34	32	16					1	
	35–49	39	7	< 0.001	2.78	1.02–7.60	0.08	2.66	0.88-8.00
	50–64	62	9	< 0.001	3.44	1.37–8.65	< 0.001*	4.21	1.45–12.25
	≥ 65	58	7	< 0.001	4.14	1.54–11.12	< 0.001*	4.91	1.60-15.03
Sex									
	Male	110	16	0.06	1.95	0.97–3.93			
	Female	81	23		1				
Residence									
	Rural	96	24	0.20	0.63	0.31–1.27	0.30	0.66	0.29–1.47
	Urban	95	15		1				
Education status									
	No formal education	54	9	0.89	1.07	0.40–2.84			
	Primary level	31	9	0.34	0.61	0.22–1.67			
	Secondary level	50	11	0.66	0.81	0.31–2.07			
	College and higher level	56	10		1				
Occupation									
	Farmer	50	11	0.38	1.94	0.43–8.74			
	House wife	29	5	0.28	2.48	0.47–12.97			
	Government employee	57	11	0.29	2.22	0.49–9.93			
	Private worker	48	9	0.28	2.28	0.49–10.53			
	Other	7	3		1				
Stage of the disease									
	Stage one	31	21		1			1	
	Stage two	32	4	< 0.001	5.41	1.66–17.59	< 0.001	5.99	1.67–21.43
	Stage three	32	3	< 0.001	7.22	1.95–26.69	< 0.001	6.49	1.63–25.87
	Stage four	39	4	< 0.001	6.60	2.05–21.25	< 0.001	6.36	1.82–22.26
	Stage five	57	7	< 0.001	5.51	2.11–14.41	< 0.001	5.48	1.99–15.09
Comorbid condition									
	Absent	44	19		1			1	
	Present	147	20	< 0.001	3.17	1.55–6.47	< 0.001*	3.53	1.55–8.06
Dialysis therapy									
	Absent	143	30		1				
	Present	48	9	0.78	1.11	0.49–2.52			
Practitioner specialty									
	General physician	107	20	0.58	1.21	0.60–2.41			
	Internist	84	19		1				

* Statistically significant AOR adjusted odds ratio COR crude odds ratio CI confidence interval

## Discussion

This study is the first of its kind, to our knowledge, that evaluates the magnitude of multiple drug use and determinates of vulnerability among inpatients with CKD in Ethiopia. In this study, males comprised the majority of participants, which is similar with findings from a study conducted at tertiary care hospital in Brazil [3] and a systematic review [20]. The higher prevalence of CKD in males may be attributed to factors associated with their lifestyle, such as chronic alcoholism, prolonged smoking, poor dietary habits, insufficient exercise, and poor healthcare-seeking behavior that exposes them to chronic diseases [24]. Regarding CKD stages, most participants in this study were classified under CKD stage 5, similar to a previous study in India [25]. However, another study reported that the majority of respondents were categorized under Stage 3 [26]. This discrepancy might be due to several factors. The demographic and clinical characteristics of study populations vary significantly. For example, our study population had a higher prevalence of risk factors such as hypertension, diabetes, or cardiovascular diseases, which can accelerate the progression of CKD. In contrast, studies reporting higher proportions of patients in earlier CKD stages have included populations with different risk profiles. Differences in healthcare access and the timing of CKD diagnosis can lead to variations in the stage distribution. In regions with more accessible and comprehensive healthcare systems, CKD might be detected and managed earlier, resulting in a higher proportion of patients in the early stages of the disease. In contrast, delayed diagnosis and limited access to healthcare services could result in a higher prevalence of advanced CKD stages [27]. Moreover, variations in the diagnostic criteria and laboratory methods used to classify CKD stages can contribute to differences across studies. Differences in the measurement of serum creatinine and the estimation of glomerular filtration rate can influence the stage at which CKD is identified. Finally, differences in study design, including sample size, inclusion criteria, and study settings, can impact the distribution of CKD stages observed.

Our study found that a high proportion of CKD inpatients received a minimum of five drugs. This finding aligns with prior studies, which have also reported an increased rate of multiple drug use among patients with CKD, with minimal differences in percentages [3, 9, 28, 29].The observed differences in the magnitude of multiple drug use across various studies may be due to variations in the methods used; different studies may employ varied methodologies, including different definitions of multiple drug use, leading to discrepancies in reported prevalence. A systematic review reported that over 80% of studies utilized various numerical thresholds to define polypharmacy, while the remaining studies adopted alternative definitions tailored to the specific care context or based on other descriptive criteria [30]. Another factor is the number of correlated complications or comorbidities. The presence of multiple comorbid conditions often necessitates the use of several medications. Hence, populations with higher rates of comorbidities, such as hypertension, diabetes, or cardiovascular diseases, are more likely to have higher levels of multiple drug use. This aligns with our findings where patients with a greater number of complications tended to be on more medications. Additionally, healthcare access and practices can significantly influence the extent of multiple drug use. Differences in healthcare systems, prescription practices, and patient adherence to prescribed treatments can also contribute to the observed variations. For instance, regions with better healthcare access and more stringent prescription practices might report lower levels of multiple drug use compared to areas with less access to healthcare and more liberal prescription practices.

In our study the incidence of multiple drug use was increasing among patients with progressed CKD Stages compared to CKD stage 1. This finding was similar to a previous study [9]. Patients in advanced stages of CKD typically have more severe symptoms and complications, requiring more extensive pharmacological management. This could explain why studies with a higher proportion of patients in later CKD stages report higher levels of multiple drug use. A significant proportion of participants in our study were classified under CKD Stage 5, necessitating more complex treatment regimens [31, 32]. The current study findings is higher compared to a systematic review that included findings from Europe, North America, and Asia [20]. The possible reason for this difference might be the better practice of clinical pharmacists in developed countries, where they collaborate with other healthcare professionals to provide medical treatment to patients with renal impairment, and the adoption of a clinical decision support system that enhances kidney-related prescribing of drugs [33, 34]. The average number of drugs used in our study was six, comparable to studies conducted in Japan, Germany, and France [9, 19, 35]. This high incidence of multiple drug use is concerning, especially in individuals with CKD, who are at greater risk to adverse effects due to the kidney’s vital role in drug metabolism [36].

Regarding specific drugs in our study, diuretics and angiotensin converting enzyme inhibitors were the most commonly used drugs, consistent with prior investigations [3, 31, 37–39]. Deprescribing is important for detecting and discontinuing unnecessary drug. It can be described as “the systemic process of finding and terminating drugs in situations where current or potential adverse effects surpass existing or potential benefits” [17, 40, 41].

Results on medication prescriptions between patients on dialysis and those not on dialysis of this study reveal significant differences in medication prescriptions between CKD patients who are on dialysis and those who are not which is supported by previous study [42]. This analysis provides valuable insights into prescribing patterns and underscores the varied therapeutic approaches based on patients’ dialysis status. Furosemide emerged as the most frequently prescribed medication, reflecting its critical role in managing fluid balance in CKD patients. It was prescribed to 50.9% of patients on dialysis and an impressive 99.4% of patients not on dialysis, resulting in an overall prescription rate of 87.4%. This high prevalence underscores furosemide’s importance in controlling symptoms of fluid overload [43], which is particularly relevant for patients with advanced CKD who are at risk of fluid retention. Enalapril, an angiotensin-converting enzyme inhibitor, was the second most commonly prescribed medication, with 43.8% of patients on dialysis and 95.3% of those not on dialysis receiving it. The total prescription rate of 85.6% highlights enalapril’s role in managing hypertension and proteinuria in CKD patients. Its high usage among patients not on dialysis suggests its efficacy in early CKD stages, where it helps in slowing disease progression and controlling blood pressure [44]. In contrast, spironolactone, a potassium-sparing diuretic, was prescribed to only 8.8% of patients on dialysis compared to 55.5% of those not on dialysis, resulting in an overall rate of 43.9%. This discrepancy may be due to the risk of hyperkalemia associated with spironolactone [45], which can be exacerbated in patients with impaired renal function. Cimetidine, a histamine H2-receptor antagonist, was prescribed to 15.8% of patients on dialysis and 50.9% of those not on dialysis, indicating its use for managing gastrointestinal issues. Insulin was used in 12.3% of patients on dialysis and 34.7% of those not on dialysis, reflecting its importance in managing diabetes, a common comorbidity in CKD patients. Hydrochlorothiazide was not prescribed to any patients on dialysis but was used by 53.2% of those not on dialysis. This difference likely reflects the limited efficacy of thiazide diuretics in advanced CKD [46], where they are less effective due to decreased renal function. Medications such as nifedipine, aspirin, and atorvastatin showed varied usage, with prescription rates indicating their roles in managing hypertension, cardiovascular disease, and hyperlipidemia. Metformin, used primarily for diabetes management, was prescribed only to patients not on dialysis, likely due to concerns about its use in renal impairment [47]. Overall, these findings illustrate the complexity of managing CKD and highlight the importance of tailoring medication regimens to the patient’s dialysis status. The variation in prescription patterns reflects the need to balance efficacy, safety, and potential risks associated with renal impairment. Future research could further explore the outcomes associated with these prescribing practices to optimize treatment strategies for CKD patients.

Regarding the nephrological procedures, nearly all patients at stage 5 in this study were on dialysis, contrary to a study where the majority of participants received conservative therapy [26]. This variation may be due to the fact that the current study included a larger proportion of patients with CKD Stage 5 than the above study.

In the current study, we also classified drug therapies into three phenotypic classes and found that the majority of participants were on multidrug therapy. This finding is significant as it highlights the complexity of managing CKD, a condition often accompanied by multiple comorbidities requiring extensive pharmacological interventions. This high prevalence is consistent with existing literature, indicating that as CKD severity progresses, patients are more likely to be prescribed multiple medications [12]. This may be due to the need to manage not only the primary condition but also associated complications. The use of multiple drugs, although necessary, increases the risk of drug-drug interactions and adverse effects, which requires careful management and monitoring by healthcare providers [48].

The present study also explored the determinates of vulnerability to multiple drug use, including older age, the presence of comorbid conditions, and CKD stage. In our study, patients over 50 years old were more vulnerable to multiple drug use than younger age groups. This finding highlights the increasing risk of polypharmacy as patients age, likely due to the accumulation of chronic conditions that necessitate more complex therapeutic regimens. This underscores the need for careful management of medications for older CKD patients to avoid potential complications arising from polypharmacy [49]. Healthcare for older adults often results in the use of multiple drugs, and special attention should be given to managing multidrug regimens, considering challenges such as consulting different healthcare professionals, physical limitations, and cognitive impairment [50, 51]. Furthermore, a significant drawback of multiple drug use is its impact on older adults’ adherence to their medications [52]. Another factor contributing to vulnerability to multiple drug use is the presence of comorbid conditions, which aligns with findings from previous studies [3]. This is likely because the presence of comorbidities and complications increases the number of drugs required [53]. Additionally, a large number of the participants in this study had comorbidities and complications, which might have contributed to the use of multiple drug therapies. Comorbid illnesses such as lipid disorders, diabetes mellitus, and hypertension are widely recognized as causes for the onset and worsening of CKD [54]. Generally the rising incidence of multiple drug use correlates with aging and the presence of multiple medical conditions [55, 56]. The other significantly associated factor in our study is stage of CKD. Patients in stages two through five of CKD are at a higher risk of multiple drug use compared to those in stage one. These findings align with previous research [9]. This may be due to the fact that as CKD progresses, patients often require a broader range of medications to control associated conditions, prevent further kidney damage, and manage symptoms. Additionally, patients in advanced stages of CKD may require medications specifically aimed at slowing the progression of kidney damage, managing complications like anemia or bone mineral disorders, and addressing symptoms related to declining kidney function [57].

A multidisciplinary approach involving regular review by pharmacists can be very beneficial for patients with acute illnesses requiring hospitalization. Pharmacists can help ensure the appropriate selection and dosing of medications, manage drug interactions, and provide patient education. This can improve medication safety and efficacy, as well as reduce the risk of adverse effects. Additional monitoring by physicians is also crucial. Regular monitoring helps in assessing the effectiveness of the treatment, adjusting dosages, and managing any side effects or complications that may arise. Combining the expertise of pharmacists with that of physicians and other healthcare professionals ensures comprehensive care and can lead to better patient outcomes.

Our study emphasizes the critical need for individualized patient care in managing CKD, where the benefits of multiple drug use must be carefully balanced against the potential risks, particularly in a population already vulnerable due to compromised kidney function. The complexity of managing CKD with multiple medications not only increases the risk of adverse drug interactions but also underscores the importance of personalized treatment strategies. This finding highlights the necessity for ongoing research aimed at optimizing drug regimens for CKD patients to reduce potential risks while enhancing therapeutic outcomes. Such an approach will ensure that treatment plans are both effective and safe, tailored to the unique needs of each patient.

## Conclusion

Chronic kidney disease patients had a high rate of multiple drug use, with older age, advanced CKD stages, and the presence of comorbid conditions identified as determinates of vulnerability. Healthcare providers should be particularly attentive to patients who are older or have comorbidities, considering the potential nephrotoxic effects of frequently used medications, such as furosemide, enalapril, amlodipine, spironolactone, cimetidine, and others. These findings highlight the importance of cautious prescribing practices to mitigate the risk of further kidney damage. Our findings provide crucial insights for promoting rational prescribing of drugs.

## Data Availability

All the necessary data is included in the manuscript. On request, the corresponding author can give any additional details.

## Abbreviation

CKD: Chronic kidney disease
